# Reduced healthcare utilization following successful hepatitis C virus treatment in HIV‐co‐infected patients with mild liver disease

**DOI:** 10.1111/jvh.12484

**Published:** 2015-10-29

**Authors:** P. Padam, S. Clark, W. Irving, R. Gellissen, E. Thomson, J. Main, G. S. Cooke

**Affiliations:** ^1^ Division of Infectious DiseasesImperial College LondonLondonUK; ^2^ NIHR Biomedical Research Unit in Gastroenterology and the LiverUniversity of NottinghamNottinghamUK; ^3^ Department of HepatologyImperial College NHS TrustLondonUK; ^4^ MRC Centre for Virus ResearchUniversity of GlasgowGlasgowUK; ^5^ Department of MedicineImperial College LondonLondonUK

**Keywords:** hepatitis C, healthcare utilization, HIV

## Abstract

New direct‐acting antivirals (DAA) for hepatitis C virus (HCV) infection have achieved high cure rates in many patient groups previously considered difficult‐to‐treat, including those HIV/HCV co‐infected. The high price of these medications is likely to limit access to treatment, at least in the short term. Early treatment priority is likely to be given to those with advanced disease, but a more detailed understanding of the potential benefits in treating those with mild disease is needed. We hypothesized that successful HCV treatment within a co‐infected population with mild liver disease would lead to a reduction in the use and costs of healthcare services in the 5 years following treatment completion. We performed a retrospective cohort study of HIV/HCV‐co‐infected patients without evidence of fibrosis/cirrhosis who received a course of HCV therapy between 2004 and 2013. Detailed analysis of healthcare utilization up to 5 years following treatment for each patient using clinical and electronic records was used to estimate healthcare costs. Sixty‐three patients were investigated, of whom 48 of 63 (76.2%) achieved sustained virological response 12 weeks following completion of therapy (SVR12). Individuals achieving SVR12 incurred lower health utilization costs (£5000 per‐patient) compared to (£10 775 per‐patient) non‐SVR patients in the 5 years after treatment. Healthcare utilization rates and costs in the immediate 5 years following treatment were significantly higher in co‐infected patients with mild disease that failed to achieve SVR12. These data suggest additional value to achieving cure beyond the prevention of complications of disease.

**Abbreviations**
BHIVA: British HIV Association
HCV: hepatitis C virus
NHS: National Health Service
RBV: ribavirin
SVR: sustained virological response
UKCHIC: UK collaborative HIV cohort
USS: ultrasound scans

## Introduction

Hepatitis C virus (HCV) is estimated to have infected over 170 million people worldwide, accounting for 3% of the global population 1. Co‐infection with HIV and HCV is common due to shared routes of transmission with the prevalence of co‐infection ranging from 9% to 30% in different settings 2, 3, 4, 5. Since the introduction of highly active antiretroviral therapies (HAART) and the reduction in mortality from malignancy and opportunistic infection, hepatic disorders have become a leading cause of death for patients with HIV in developed nations 6, 7, 8, with HCV playing a major role. HIV‐infected individuals with HCV experience more rapidly progressive fibrosis and an increased risk of cirrhosis and hepatocellular carcinoma, occurring in 25% and 1.6% of co‐infected individuals over their lifetime, respectively 9, 10.New direct‐acting antivirals (DAAs) against HCV have the potential to cure many HCV/HIV‐co‐infected patients who have not tolerated or have failed previous treatments. However, widespread access to these treatments is currently beyond existing health budgets 11 in most economies, and their initial use is likely to be limited to patients with significant fibrosis or cirrhosis 12. Strong justification of the cost benefit of treatment in patients with mild disease will be required.

Several studies have reported higher usage of healthcare services such as hospitalizations and emergency room visits in co‐infected individuals than amongst HIV‐infected patients 13, 14, 15, 16. There is some evidence that sustained virological response (SVR) in HCV‐monoinfected patients is cost‐saving 17. In contrast to HCV‐monoinfected patients, HIV/HCV‐co‐infected patients remain in secondary care even when cured and the impact on healthcare utilization has not been studied in this population.

We aimed to investigate whether HIV/HCV‐co‐infected patients who were successfully treated for mild hepatitis C had reduced usage of healthcare services and costs after completion of successful treatment.

## Materials and Methods

### Study population

**Figure 1:**
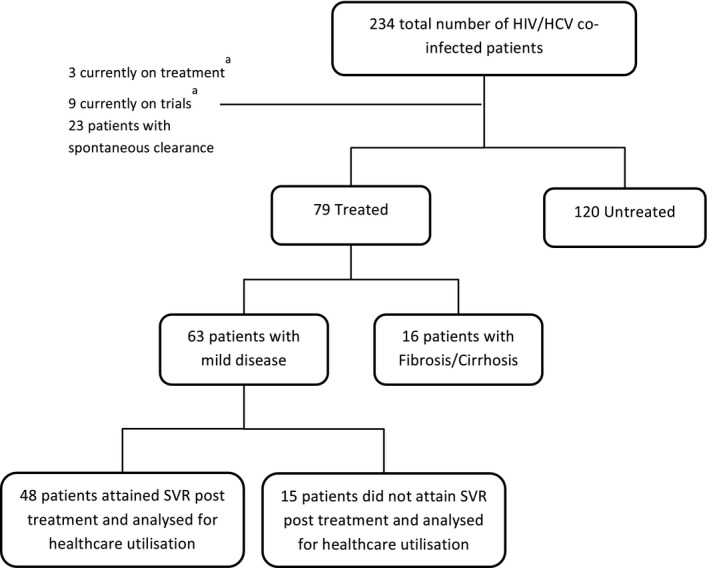
Selection of study cohort. ^a^As of March 1 2014. HCV, hepatitis C virus; HIV, human immunodeficiency virus; SVR, sustained virological response.

Patients were eligible if they attended the study clinic between 1 January 2004 and 1 March 2013. Patients included for analysis required (i) confirmed positive HIV antibody status, (ii) evidence of HCV infection, and for this study, patients being positive for HCV RNA on more than one occasion and (iii) to have received and completed at least 3 months of treatment for HCV between 1 January 2004 and 1 March 2013. This would allow us to obtain at least 1 year of follow‐up for all patients by the date of data collection, 1 March 2014. Patients were included regardless of treatment type which included pegylated interferon (PEG‐IFN, both α‐2b, Schering‐Plough or α‐2a, Roche), ribavirin (RBV) and latterly protease inhibitors (PI). Analysis was limited to patients without evidence of significant fibrosis to avoid confounding by the fact that patients with more advanced disease have greater healthcare costs and that those with most advanced disease respond less well to therapy 18, 19, 20. Patients we considered not to have significant fibrosis were those who had a fibroscan result <9.6 kPa and/or a biopsy with ISHAK stage score <2/6 in the 2 years prior to treatment. Individuals who were currently on treatment were excluded from the study. Treated patients were separated into two groups based on outcome – those who attained SVR and those that did not (non‐SVR) as shown in Fig. 1. Patients were recruited from a single centre where the majority of patients are from West London, United Kingdom (UK).

Data on patient characteristics, clinical data and healthcare utilization were collected from clinical records supplemented by electronic records for investigations and hospital attendance. Data collected independently by UK collaborative HIV cohort (UKCHIC) 21 were used to cross‐reference information from hospital databases and verify patient selection. UKCHIC is a collaboration that routinely collects data on HIV‐positive individuals who have received care at any one of the associated centres in the United Kingdom.

### Baseline characteristics

Baseline patient information for the entire HIV/HCV‐co‐infected cohort included the patient's age, gender, race, fibrosis/cirrhosis status, baseline laboratory data comprising of CD4 count, HCV genotype, HCV and HIV viral loads, fibroscan results and biopsies. Baseline analysis was then repeated for SVR and non‐SVR groups to allow comparison between cohorts once individuals were identified. For both groups, additional information on treatments given and the precise dates of treatment completion were obtained from patient records.

### Healthcare utilization

Patients with mild liver disease who received a course of PEG‐IFN and RBV +/‐ protease inhibitors in line with the British HIV Association (BHIVA) guidelines were considered for medical service utilization analysis 22. Forty‐eight patients who achieved SVR and fifteen non‐SVR patients were included. For each patient, annual resource utilization data were recorded for up to 5 years post‐treatment with year 1 starting 12 weeks following treatment cessation. Healthcare follow‐up was conducted up until 1 March 2014. Outpatient attendances, clinic visits, hospital admissions, nights spent at hospital, A&E visits, number of bloods taken, HCV viral loads, number of USS and fibroscans were recorded. Clinic visits were denoted as consultant‐led encounters at the HIV study clinic. Outpatient attendances included both planned and unplanned visits to hospital outside of regular HIV clinic sessions. In the United Kingdom, a stable HIV patient routinely receives a follow‐up every 3–6 months as mentioned in the BHIVA monitoring guidelines and will have a regular blood test at least a week prior to each clinic visit. Individuals who are not tolerating treatment or with additional complications will need more frequent visits 23. This study did not consider pharmacy costs of HIV and other drugs received during the follow‐up period, which were assumed to be the same in both SVR and non‐SVR groups.

### Statistical analysis and costs

We assessed differences in healthcare utilization by comparison of rates per‐patient year of follow‐up of each healthcare service. We followed this by calculating total utilization rates over the 5 years and compared total usage per‐patient year between SVR and non‐SVR groups. Risk ratios were then determined for non‐SVR *vs* SVR patients. Statistical significance was determined at *P* < 0.05 estimated using two‐sided Student's *t*‐tests. To fully assess the benefits of attaining an SVR, we used mean costs as the factor of comparison. Healthcare service costs were obtained from the Department of Health using most recent reference costs, 2012–2013 24. Unit costs were found to be as follows: outpatient attendance including both visit and average cost of outpatient procedure £240, consultant‐led HIV clinic visit £354, hospital admission £693, night stays £1489 per night, A&E visit £115, bloods £27, HCV viral load £75, diagnostic tests (fibroscans and ultrasound scans) each at £92. Total National Health Service (NHS) expenditure for SVR and non‐SVR patients during the 5‐year follow‐up period was calculated using single unit costs and utilization rates which were later compared.

## Results

### SVR vs non‐SVR characteristics

A total of 63 co‐infected patients with mild liver disease received and completed at least 3 months of antiviral therapy between January 2004 and March 2013. Table 1 shows the comparison of baseline characteristics for SVR and non‐SVR groups. Overall, 48 of 63 patients (76%) had successful treatment of which 28 patients (58%) had acute infection. Both SVR and non‐SVR groups comprised predominantly of males. The distribution of age varied between groups where the majority (30/48, 62.5%) of SVR patients were aged 45 or older, whereas (13/15) 86.6% of non‐SVR patients were 44 or lower. A higher proportion of genotype 1 patients (44/139, 32%) received treatment as opposed to only 25% (2/8) of genotype 2 and 22% (7/32) of genotype 3 patients (data not shown). Twenty‐five per cent (11/44) of patients treated for genotype 1 and (4/10) 40% of patients treated for genotype 4 failed therapy, whilst all individuals treated for genotypes 2 and 3 had successful outcomes.

**Table 1:** Baseline characteristics of the SVR *vs* non‐SVR cohorts

	SVR *n* = 48	Non‐SVR *n* = 15
	*n* (%)	*n* (%)
Gender		
Female	1 (2.1)	0
Male	47 (97.9)	15 (100)
Age (years)		
Mean, standard deviation	46, 8.23	41, 6.65
Median, range (min, max)	46, 38 (29,67)	40, 28 (30,58)
Distribution (years)		
25–34	3 (6.25)	2 (13.3)
35–44	15 (31.3)	11 (73.3)
45–54	24 (50)	1 (6.7)
55–64	5 (10.4)	1 (6.7)
>65	1 (2.1)	0
Race/Ethnicity		
White	40 (83.3)	13 (86.7)
Black	2 (4.2)	0
Asian	5 (10.4)	1 (6.7)
Other	1 (2.1)	1 (6.7)
HCV Status		
Acute	28 (58.3)	8 (53.3)
Chronic	20 (41.7)	7 (46.7)
HCV genotype*		
Genotype 1	33 (68.8)	11 (73.3)
Genotype 2	2 (4.2)	0
Genotype 3	7 (14.6)	0
Genotype 4	6 (12.5)	4 (26.7)
HIV viral load† (copies/mL)		
<50	36 (75)	13 (86.7)
≥50	12 (25)	2 (13.3)
CD4 count† (copies/μL)		
101–500	19 (39.6)	3 (20)
501–1000	27 (56.3)	11 (73.3)
>1000	2 (4.2)	1 (6.7)
Median duration of follow‐up (years)	4	5

HCV, hepatitis C virus; HIV, human immunodeficiency virus; SVR, sustained virological response. *Genotype for which treatment given. ^†^As of March 1st 2014.

### Health service utilization post‐treatment

Table 2 shows the annual healthcare utilization rates per‐patient for each of the services measured post‐treatment. The median duration of follow‐up was 4 and 5 years for SVR and non‐SVR groups, respectively. Compared to those with a SVR, non‐SVR patients had higher annual utilization rates for five of the nine measured healthcare services (hospital admissions, fibroscans, USS, clinic visits and outpatient attendances) in the 5 years following treatment. A&E and night stays did not show a significant difference between both cohorts, and this is due to the relatively low utilization rates seen during each year of the follow‐up.

**Table 2:** Annual post‐treatment healthcare utilization of SVR *vs* non‐SVR patients

Healthcare service	Years after treatment									
	Year 1*		Year 2		Year 3		Year 4		Year 5	
	SVR	Non‐SVR	SVR	Non‐SVR	SVR	Non‐SVR	SVR	Non‐SVR	SVR	Non‐SVR
Outpatients attendances	1.81	4.20	1.77	3.07	1.3	3.29	1.23	2.31	1.3	2.44
Clinic visits	1.4	2.40	1.2	1.93	0.95	2.00	0.97	1.46	0.85	2.11
Hospital admissions	0.04	0.13	0.07	0.29	0.08	0.50	0.03	0.38	0	1
Nights stayed in hospital	0.02	0	0.02	0.21	0.05	0.29	0.1	0	0	0.11
A&E	0	0.7	0.07	0.07	0.08	0.07	0	0	0	0
Blood draws	2.48	2.93	2.2	2.21	1.9	2.43	1.6	1.54	1.65	2.11
HCV viral loads	1.19	1.47	1.09	0.86	0.98	1.07	0.67	0.54	0.45	0.56
USS	0	0.2	0.05	0.5	0	0.43	0	0.23	0.05	0.33
Fibroscans	0	0.2	0	0.29	0	0.36	0.1	0.31	0.05	0.33

HCV, hepatitis C virus; SVR, sustained virological response; USS, ultrasound scans. Rates for each year given per‐patient. *Beginning 12 weeks from treatment completion (SVR12).

**Figure 2:**
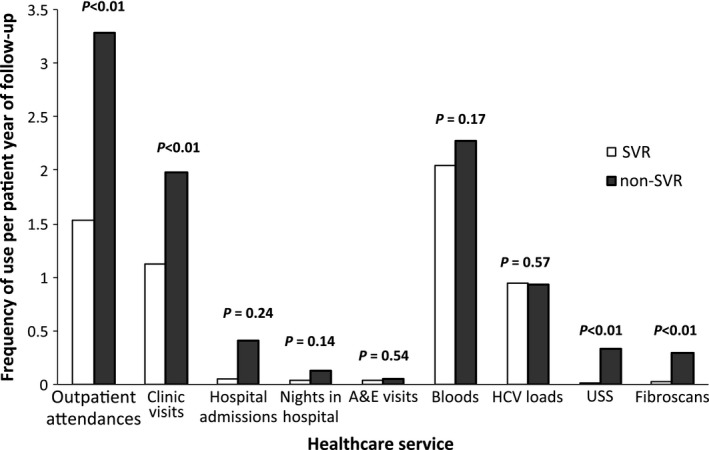
Frequency of healthcare utilisation per patient‐year. Total follow‐up years: 182 SVR, 65 non‐SVR. Statistical significance detected at *P* < 0.05. HCV, hepatitis C virus; SVR, sustained virological response; USS, ultrasound scan; A&E, accident and emergency.

We then investigated how the use of these services varied between groups over the follow‐up period. Figure 2 shows the utilization rates per person year over the initial 5 years upon treatment completion. Outpatient attendances were significantly higher in non‐SVR patients (3.3 visits per‐patient year) when compared to 1.5 for SVR patients (*P* = 0.0022). Likewise, significant differences were seen in the average number of clinic visits over the course of the study with SVR and non‐SVR using the service 1.1 and 2 times per‐patient year, respectively (*P* = 0.0018). Those not achieving SVR were more likely to have an ultrasound scan and a fibroscan in the initial 5 years following treatment with a relative risk (RR) of 14.93 (95% CI, 4.95–45.04, *P* < 0.0001) and 10.40 (95% CI, 3.99–27.14, *P* < 0.0001), respectively. There was an associated increase in relative risk with the use of all listed healthcare services in the absence of SVR; however, results for hospital admissions and A&E visits did not prove statistically significant.

### Resource costs post‐treatment

To ascertain the financial benefits of attaining a SVR, we calculated total costs for resource utilization. Table 3 shows the estimated costs per service over the 5 year follow‐up for both cohorts. Patients who failed treatment incurred higher healthcare utilization costs than those successfully treated, and this trend is apparent in all measured services. Healthcare costs for non‐SVR subjects totalled £2155 per‐patient year compared to only £1000 for SVR patients. Over a 5‐year period, utilization costs would therefore amount to £10 775 and £5000 for non‐SVR and SVR patients, respectively. As very few A&E visits were observed throughout the study for both groups, the economic impact on reducing admissions is small. The greatest disparities in costs between the cohorts were found in ultrasounds amounting to a 93% difference between groups.

**Table 3:** Healthcare costs per‐patient per year for the SVR *vs* non‐SVR cohorts

	HIV/HCV											
	Follow‐up (years)	Outpatient attendances	Clinic visits	Hospital admissions	Night stays	A&E visits	Bloods	HCV viral loads	USS	Fibroscans	Total	Cost per‐patient per year
SVR (*n* = 48)	182	£67 200	£72 216	£6237	£10 423	£690	£100 71	£12 975	£276	£2024	£182 112	£1000
Non‐SVR (*n* = 15)	65	£51 120	£45 666	£18 711	£11 912	£345	£3996	£4575	£2024	£1748	£140 097	£2155

Unit costs obtained from the Department of Health. Costs displayed in GBP. HCV, hepatitis C virus; USS, ultrasound scans; SVR, sustained virological response.

## Discussion

The study found that within a HIV/HCV‐co‐infected population with mild disease, unsuccessful treatment is associated with significantly higher costs (£1155 more per year) of healthcare utilization per‐patient following HCV therapy than those who were successfully treated. Outpatient and clinic attendances were higher in non‐SVR patients when compared to those achieving SVR (£766/£1000, 77%) in comparison with non‐SVR group (£1489/£2155, 69%). A greater proportion of costs were attributable to hospital admission in those who failed treatment. Of the minority of patients who failed treatment, none had developed severe fibrosis or cirrhosis throughout the duration of the study and so healthcare analysis was not influenced by progression of disease. No significant difference was seen in the number of bloods taken between both cohorts reflecting the fact that both cohorts would continue to be monitored for their HIV irrespective of treatment response. It is also worth noting that although the total cost spent on inpatient services (hospital admissions and night stays) was higher for non‐SVR patients (£471 per‐patient) than SVR patients (£91 per‐patient), the rates for inpatient services were very low for both groups (Table 2).

This study adds to the growing literature on the consequences of successful treatment of HCV, which inform our understanding of cost‐effectiveness. This is the first study to explore the impact of successful treatment in individuals with HIV co‐infection and mild disease, an important group who may play a key role in ongoing transmission of infection if not being prioritized for treatment based on liver fibrosis. Recent work has explored the benefits of treatment in HCV‐monoinfected patients 17, 25, and a different cohort from the one investigated here not least because HCV‐monoinfected individuals with mild disease can potentially be discharged from secondary care after successful treatment. A UK study reported a thirteen‐fold difference in costs between SVR and non‐SVR patients with chronic hepatitis C limited to those with genotype 1 17. The healthcare services measured in the study were similar to those in our analysis with the addition of CT and MRI scans in place of fibroscans. Total costs incurred for SVR patients per year amounted to £54 in comparison with £506 for those who failed treatment 17. A US study calculated post‐treatment healthcare costs in monoinfected HCV patients to be 1.6 times higher in non‐SVR subjects than those with successful outcomes upon treatment 25. That study considered those with cirrhosis grouped alongside patients with little or no liver disease and is thus not directly comparable to our findings.

The study has several limitations. Outpatient attendances, hospital admissions and A&E visits were recorded based on all causes; we did not attempt to differentiate whether utilization was due to liver related events caused by HCV. Comorbidities such as obesity and diabetes were not studied in detail and some change in use of services may reflect non‐hepatic consequences of infection. The design of this study was intended to minimize the potential for confounding in findings as a result of the lower SVR rates seen in those with progressive fibrosis 20. However, we cannot exclude the fact that there may be patients within the study where the extent of liver disease may be underestimated by previous fibroscans and biopsies. Whilst the largest study of this population to date, the numbers of patients included are relatively small and the findings require confirmation in other studies. In particular, data from a larger number of centres would be helpful to establish if the data are representative of wider practice (for example, the number of visits even in those patients achieving SVR is greater than would be expected under national guidelines). The SVR rates seen in this study are relatively high (76%), and this likely reflects a significant number of patients receiving treatment for acute infection. Although different from the HCV mono‐infection period, this is quite typical of practice in co‐infection.

Finally, the study relied on data from electronic databases and patient records, and it may be that all patient‐related data were not captured (for example, attendances at other centres or in primary care). It is not possible to estimate the extent of this issue within this study although it may lead to an underestimate of the changes in health utilization. In addition, this study did not consider pharmacy costs of HIV and other drugs received during the follow‐up period, which too will have contributed to the overall healthcare costs for both cohorts.

The results from our study add to existing data informing the cost‐effectiveness of antiviral therapies. Whilst we cannot yet know whether successful DAA therapy will have the same benefits, it is likely that the benefits will be similar but further, ideally larger, studies are required. It is possible that the difference between SVR and non‐SVR groups will change with a longer period of follow‐up, but it is likely that there will be greater divergence as disease progression in the non‐SVR group will require more frequent monitoring.

In conclusion, this study was the first to compare the impact of successful HCV treatment on healthcare utilization in a HIV/HCV‐co‐infected population with mild disease. Our results show significant differences in healthcare costs and utilization rates between individuals that are successfully treated for HCV compared to those failing treatment, despite the fact that they remain in secondary care.

The study provides data in addition to the known benefits of SVR in reducing the risk of cirrhosis, hepatocellular cancer, end‐stage liver disease and disease transmission 26 and adds to the evidence for cost‐effectiveness of treatment in this population.
